# Improve the Anaerobic Biodegradability by Copretreatment of Thermal Alkali and Steam Explosion of Lignocellulosic Waste

**DOI:** 10.1155/2016/2786598

**Published:** 2016-04-20

**Authors:** Muhammad Abdul Hanan Siddhu, Jianghao Li, Jiafu Zhang, Yan Huang, Wen Wang, Chang Chen, Guangqing Liu

**Affiliations:** ^1^Biomass Energy and Environmental Engineering Research Center, College of Chemical Engineering, Beijing University of Chemical Technology, Beijing 100029, China; ^2^College of Life Science and Technology, Beijing University of Chemical Technology, Beijing 100029, China

## Abstract

Effective alteration of the recalcitrance properties like crystallization of cellulose, lignin shield, and interlinking of lignocellulosic biomass is an ideal way to utilize the full-scale potential for biofuel production. This study exhibited three different pretreatment effects to enhance the digestibility of corn stover (CS) for methane production. In this context, steam explosion (SE) and thermal potassium hydroxide (KOH-60°C) treated CS produced the maximal methane yield of 217.5 and 243.1 mL/g_vs_, which were 40.0% and 56.4% more than untreated CS (155.4 mL/g_vs_), respectively. Copretreatment of thermal potassium hydroxide and steam explosion (CPTPS) treated CS was highly significant among all treatments and improved 88.46% (292.9 mL/g_vs_) methane yield compared with untreated CS. Besides, CPTPS also achieved the highest biodegradability up to 68.90%. Three kinetic models very well simulated dynamics of methane production yield. Moreover, scanning electron microscopy (SEM), Fourier transform infrared (FTIR), and X-ray diffraction (XRD) analyses declared the most effective changes in physicochemical properties after CPTPS pretreatment. Thus, CPTPS might be a promising approach to deconstructing the recalcitrance of lignocellulosic structure to improve the biodegradability for AD.

## 1. Introduction

China is the second largest maize producer worldwide. Mostly, maize is cultivated in the central and north part of China. According to crop ratio index and China statistic year book, around 400 MTs of corn stover (CS) were generated in 2012 [1]. But approximately, two-thirds of produced CS was not utilized. Farmers burnt or threw away this portion of CS, because of quick preparation of land for next crop cultivation, high input cost for collection, and cheap market prices. This practice not only caused the natural resource depletion but also stimulated many serious environmental issues on local and regional scale, for example, air pollution, global warming, climate change, and so forth [2]. Hence, it is a great responsibility standing on researcher's shoulder to develop solution for effective utilization of CS by conserving environment as well as the natural resource.

Anaerobic digestion (AD) is a complicated biological process for treatment of various types of organic wastes (municipal waste, agricultural waste, industrial waste, etc.) for generation of biogas as well as protecting our environment [3]. However, biodegradability and AD performance of organic waste depend on its characteristics. Agricultural sector is key contributor for generation of lignocellulosic waste, and the main components of this waste are cellulose, hemicellulose, and lignin. Physicochemical quantitative and qualitative properties of lignocellulosic biomass such as accessible surface area, crystallization nature, lignin polymerization, and cross linkages of cell wall should be deconstructed for improving the anaerobic digestibility. Thus, effective and moderate conditions of different pretreatment methods can be used to overcome recalcitrance nature of biomass in order to achieve effective utilization and increase the anaerobic digestibility [4].

Steam explosion (SE) is an environmental friendly method for the pretreatment of lignocellulosic biomass [5]. In SE pretreatment, lignocellulosic biomass undergoes saturated steam at high pressure for short time, and then the pressure is released suddenly. This process causes the physical disruption of lignocellulosic biomass such as rupturing the cross linkages of cell walls and transmutation of hemicellulose [6]. Thus, more efforts should be addressed to use the SE pretreatment for lignocellulosic biomass to improve the hydrolysis and anaerobic digestibility [7–9].

In the past years, alkali pretreatment has also been extensively studied to enhance the hydrolysis of lignocellulosic biomass. Overall alkali pretreatment performance is to swell and solubilize lignocelluloses with an outcome of enhancing the biodegradability. Besides, thermal alkali pretreatment of lignocellulosic biomass can effectively destroy the recalcitrance properties, while some inhibitory substances start to produce during the treatment at high temperature (>100°C) with NaOH. Some studies reported that treatment of biomass with mild thermal NaOH (lower than 100°C) was more effective way to remove lignin, enhance hydrolysis, and improve the biodegradability for AD as compared to room temperature [10–12]. However, some studies reported the issues related to high loading of NaOH like toxic to microorganisms, soil salinity, and difficulty of recycling [13]. So considering these issues, potassium hydroxide (KOH) might be preferred over NaOH, because KOH black liquor can be used as soil reclamation and as fertilizer in agriculture sector. Thus, KOH pretreatment at mild thermal might be more suitable and effective condition to enhance the hydrolysis of lignocellulosic biomass for AD.

Copretreatment of thermal KOH and SE might have more synergistic effect to overcome the recalcitrance nature of lignocellulosic biomass and improve digestibility for AD.

The goals of this work were to (1) measure the methane production potential and digestion performance of untreated, thermal KOH treated, and CPTPS treated CS; (2) determine the most effective pretreatment method to improve the CS digestibility; and (3) compare the physicochemical structure changes of CS after different pretreatments by scanning electron microscopy (SEM), Fourier transform infrared (FTIR), and X-ray diffraction (XRD) analyses.

## 2. Materials and Methods

### 2.1. Substrate and Inoculum

CS was obtained from Deqingyuan Company's farm, Beijing, China. CS was chopped by a 9SC-360 kneading machine (Shuncheng, China). Then CS was air-dried and bigger particle size was manually cut down by scissors in the length range of 1.5–2 cm. The dried samples of CS were kept in airtight plastic bags and stored at 4°C for later use. The inoculum for this study was the effluent of Nanwu Biogas Plant operated in Beijing, China.

### 2.2. Thermal Potassium Hydroxide Pretreatment

Four different concentrations (0.5%, 1.0%, 1.5%, and 2.0%, W/V) of KOH were added to 1.5 L plastic boxes. After this, CS was soaked into aqueous KOH solutions to increase moisture content up to 90% by using (1) [14]. Then the alkaline pretreatment was carried out at 60°C for 12 h, because low temperature (below 80°C) should be more preferable since production of inhibitory substances may occur during long period of thermal pretreatment at high temperature [10]. Stirring of each box was carried out every 4 h for 1 min during the thermal KOH pretreatment. After completion of the thermal pretreatment, CS was squeezed and put into airtight bags and stored at 4°C for next study:

(1) $$\begin{array}{c} \text{MC}\left(\;\%\;\right)=\left(\;1-\frac{\text{dry weight of CS}}{\text{weight of CS}+\text{water added}}\;\right)\times 100. \end{array}$$

### 2.3. Copretreatment of Thermal Potassium Hydroxide and Steam Explosion (CPTPS)

CPTPS was two-step copretreatment of CS with thermal KOH and SE. In this process, CS was pretreated with 0.5% and 1.5% KOH at 60°C for 12 h and then steam-exploded at 1.2 MPa for 10 min. After completing this pretreatment, CS was packed in two airtight bags and kept at 4°C. One bag was used for anaerobic digestion, while sample of second bag was dried at 60°C for 3 days for physicochemical analysis.

### 2.4. Batch Anaerobic Digestion Tests

Anaerobic batch digestion tests of untreated and treated CS were conducted in 1-L serum bottles. All the anaerobic digestion setup was triplicated [15]. All the digesters were fed 1 : 1 ratio of substrate to inoculum (*S*/*I*) on the basis of VS [16]. Working volume of each digester was adjusted to 500 mL by addition of deionized water. Each digester headspace was flushed with 99.0% pure argon for 4 min to ascertain the anaerobic atmosphere. Then rubber stopper and screw cap were fixed to digesters for sustaining the anaerobic conditions, before being placed in an incubator for running AD at 37°C for 28 days. Two parallel blank digesters containing the same quantity of inoculum were run in order to correct the methane production. All digesters were shaken twice manually each day for 1 min during the digestion time.

### 2.5. Analytical Methods

APHA standard methods were used to determine the total solid (TS), volatile solid (VS), and fixed solid (FS), of CS and inoculum [17]. Total ammonia nitrogen (TAN) and total alkalinity (TA) in the effluent were measured according to Li et al.'s reported methods [18]. Elemental compositions (C, H, N, and S) of CS and inoculum were measured by elemental analyzer (Vario Elcube, Germany). The oxygen content of CS on VS basis was estimated by assumption of C + H + O + N = 99.5% [19]. The pH of each digester was measured by a le438 pH electrode (Mettler Toledo, USA) [20]. Van Soest et al.'s reported method for measuring lignocellulosic content (cellulose, hemicellulose, and lignin) of CS was used [21].

Daily biogas production was measured by testing the pressure in the headspace of each digester. Pressure in the headspace was determined by a 3151 WAL-BMP-Test system pressure gauge with the precision of 0.1% (based on the gauge range) manufactured by WAL Mess- und Regelsysteme GmbH, Germany [22]. After this, daily biogas production was calculated by using the following equation [23]:

(2) $$\begin{array}{c} V_{\text{biogas}}=\Delta P\times V_{\text{head}}\times \frac{C}{\left(R\times T\right)}, \end{array}$$

where *V*_biogas_ stands for daily biogas volume (L), Δ*P* represents absolute pressure difference (MPa), *V*_head_ is volume of the headspace (L), *C* expresses gas molar volume under standard condition (22.4 L/mol), *T* stands for absolute temperature (K), and *R* is universal gas constant (8.314 J/K/mol).

Biogas composition (CH_4_, H_2_, and CO_2_) was measured by a 7890A gas chromatograph (Agilent, USA) equipped with a thermal conductivity detector. A Hitachi S-4700 (Japan) scanning electron microscopy (SEM) at a magnification of 500x was used to investigate the morphology of untreated and treated CS. Fourier transform infrared (FTIR) technique was applied to record the spectra of untreated and treated CS by using a Nicolet 6700 FTIR spectrophotometer (Thermo Fisher Scientific, Waltham, MA) equipped with a DLaTGS detector in the range of 4000–400 cm^−1^. Fine power of 2 mg of untreated and treated CS was mixed with 100 mg of KBr and compacted into pellets for examination. Change in crystallinity of CS before and after the pretreatment was compared by using X-ray diffraction (XRD) analysis. Germany Bruker D8-Advance with Cu K*α* radiation was used to analyze the crystallinity features of CS. Scans were obtained from 2*θ* of 5–60° at a rate of 5°/min and the index (CrI) was calculated by following equation of Sun et al. [24]:

(3) $$\begin{array}{c} \text{CrI}\left(\;\%\;\right)=\left(\;\frac{I_{002}-I_{18^{{}^{\circ}}}}{I_{002}}\;\right)\times 100, \end{array}$$

where CrI refers to crystallinity index (%), *I*_002_ expresses the maximal scattered intensity on the 002 lattice plane at main peak around 22°, and *I*_18°_ stands for amorphous zone scattered intensity at 2*θ* of 18°.

### 2.6. Theoretical Methane Yield (TMY)

Theoretical methane yield (TMY) on the basis of different organic elements presented in the CS was calculated by Buswell formula as shown in (4) and (5) [25]. One has

(4) CnHaObNc+n−a4−b2+3c4H2O⟶n2+a8−b4−3c8CH4+n2−a8+b4+3c8CO2+cNH3

(5) TMYmL CH4g VS=22.4×1000×n/2+a/8−b/4−3c/812n+a+16b+14c.

### 2.7. Biodegradability (*B*_*d*_)

Anaerobic biodegradability (*B*_*d*_) of CS during the digestion was calculated on the basis of experimental methane yield (EMY) and theoretical methane yield (TMY), and the methodology was based on Elbeshbishy formula [26]:

(6) $$\begin{array}{c} B_{d}\left(\;\%\;\right)=\frac{\text{EMY}}{\text{TMY}}\times 100. \end{array}$$

### 2.8. Kinetics Analysis

Three different models (first-order, modified Gompertz, and Cone) were applied to simulate and understand the kinetics of cumulative methane yields [27].

Fist-order model is as follows:

(7) $$\begin{array}{c} B=B_{0}\left[\;1-\exp\left(\;-kt\;\right)\;\right]. \end{array}$$

Modified Gompertz model is as follows:

(8) $$\begin{array}{c} B=B_{0}\exp\left\{\;-\exp\left[\;\frac{\mu_{m}e}{B_{0}}\left(\;\lambda -t\;\right)+1\;\right]\;\right\}. \end{array}$$

Cone model is as follows:

(9) $$\begin{array}{c} B=\frac{B_{0}}{\left[1+{\left(kt\right)}^{-n}\right]}, \end{array}$$

where *B* represents the cumulative methane yield (mL/g_vs_); *B*_0_ is the ultimate methane yield (mL/g_vs_); *k* stands for the first-order rate constant (1/d); *k* is the first-order rate constant; *μ*_*m*_ refers to the maximum methane production rate (mL/g_vs_/d); *λ* means the lag phase time (d); *e* is equal to 2.72; *t* represents the anaerobic digestion time (d); and *n* is for shape factor (dimensionless).

## 3. Results and Discussion

### 3.1. Characteristics of CS and Inoculum

Characterization of the CS and inoculum has been illustrated in Table 1. CS was comprised of TS and VS content of 93.99% and 90.02%, respectively, whereas VS to TS proportion was 95.77%, which indicated high organic content in CS and high biogas production potential. Lignocellulosic constituent of CS was comprised of 44.32% of cellulose, 33.54% of hemicellulose, and 8.28% of lignin, while more than 75% of lignocellulosic content indicated a slow hydrolysis, long digestion time, and less volume of biogas yield achievable. On the elemental composition basis of CS, the organic content was formulated as C_43.82_H_69.88_O_33.77_N, and TMY of CS was calculated to be 425.1 mL CH_4_/g_vs_ by using (5).

### 3.2. Anaerobic Digestion of Thermal Potassium Hydroxide Treated Corn Stover

**Figure 1:**
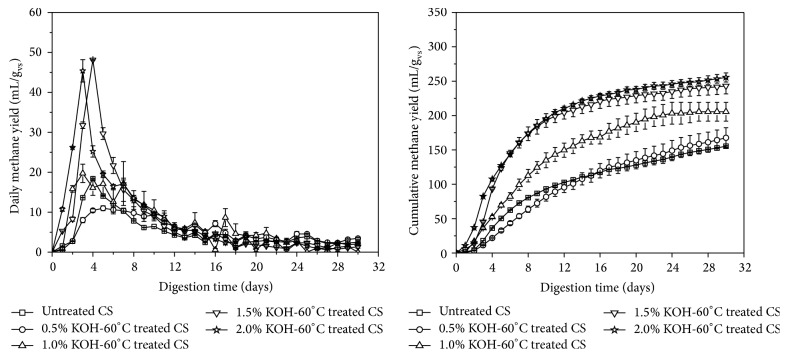
Methane yields of untreated and KOH-60°C treated CS. Error bars were obtained based on *n* = 3.

Thermal KOH treatment effects on daily and cumulative methane yields were presented in Figure 1. Highest peaks of daily methane yield of thermal KOH treated and untreated CS were appeared within the first five days of digestion, while maximum daily methane yield of thermally 60°C of 0.5%, 1.0%, 1.5%, and 2.0% KOH treated CS was 11.0 ± 0.60, 19.7 ± 2.30, 48.1 ± 0.08, and 45.4 ± 2.80 mL/g_vs_, respectively. However, no significant improvement in daily maximum methane yield of thermally 60°C of 1.5% to 2.0% KOH treated CS was observed. The cumulative methane yields of 0.5%, 1.0%, 1.5%, and 2.0% thermal KOH treated CS were 167.8 ± 14.43, 205.5 ± 13.69, 243.2 ± 11.56, and 255.4 ± 6.60 mL/g_vs_, respectively, and increase in yield was observed with the increasing of KOH concentration. However, no significant enhancement in yield from thermal 1.5% KOH to 2.0% KOH treated CS was observed, while 1.5% KOH-60°C treated CS significantly improved the methane yield, 56.40%, 48.43%, and 24.17%, respectively, compared with untreated (155.4 ± 1.02 mL/g_vs_) and thermal 0.5% to 1.0% KOH treated CS. Li et al. conducted AD experiment of 1.5% KOH treated CS at 20°C and reported only 45.1% increase in cumulative methane yield with respect to untreated CS [22]. Therefore, mild thermal pretreatment is highly effective for destruction of complex nature of recalcitrance of CS to enhance the digestibility for AD.

### 3.3. Effect of Copretreatment of Thermal Potassium Hydroxide and Steam Explosion on Anaerobic Digestion of Corn Stover

**Figure 2:**
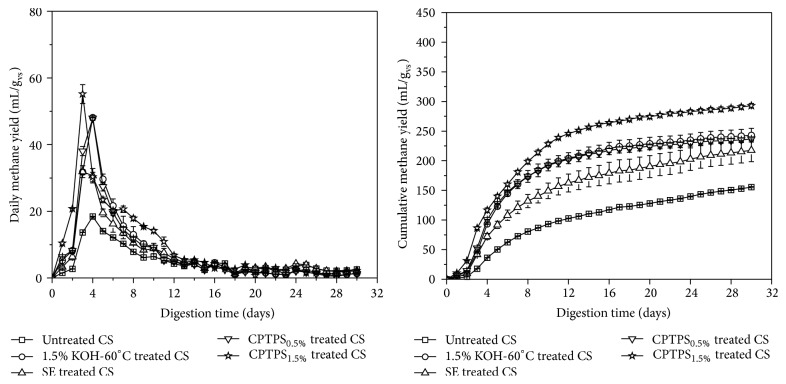
Methane yields of untreated, thermal KOH treated, SE treated, and CPTPS treated CS. Error bars were obtained based on *n* = 3.

Copretreatment of CS with thermal KOH and SE was conducted to enhance the anaerobic digestibility. The SE treated CS at 1.2 MPa for 10 min was tagged as SE, and copretreatments of 0.5% KOH-60°C or 1.5% KOH-60°C and SE were labeled as CPTPS_0.5%_ and CPTPS_1.5%_, respectively.  Figure 2 presents daily and cumulative methane yield of untreated CS and pretreatment effects on CS. Highest peaks of daily methane yield of SE and CPTPS_1.5%_ were appeared on the 3rd day and thermal 1.5% KOH-60°C and untreated CS were looked on the 4th day of the digestion, meanwhile maximum daily methane yield of CPTPS_1.5%_ and SE was 55.2 ± 2.80 and 31.9 ± 1.79 mL/g_vs_, respectively. Thus, CPTPS_1.5%_ significantly deconstructed the lignocellulosic structure of CS and start the digestion faster. Cumulative methane yields of SE, thermal 1.5% KOH-60°C, CPTPS_0.5%_, and CPTPS_1.5%_ were 217.5 ± 19.1, 243.2 ± 11.6, 236.6 ± 2.7, and 292.9 ± 3.8 mL/g_vs_, respectively. SE, thermal 1.5% KOH-60°C, CPTPS_0.5%_, and CPTPS_1.5%_ treated CS significantly improved cumulative methane yields, 39.95%, 56.40%, 52.24%, and 88.46%, respectively, compared to untreated (155.5 mL/g_vs_). But no significant variance among cumulative methane yields of thermal 1.5% KOH-60°C, SE, and CPTPS_0.5%_ was observed. Moreover, CPTPS_1.5%_ significantly enhanced 23.8% of cumulative methane yield compared with CPTPS_0.5%_. Therefore, CPTPS_1.5%_ was an effective pretreatment method to destroy the lignocellulosic complex structure among all and to improve the biodegradability for methane production.

### 3.4. Digestion Performance of Untreated and Pretreated CS

Total alkalinity (TA), total ammonia nitrogen, and biodegradability after the AD of untreated CS, 1.5% KOH at 60°C, SE, CPTPS_0.5%_, and CPTPS_1.5%_ treated CS were measured to evaluate the digestion process stability performance and presented in Table 2. Alkalinity (carbonate/bicarbonate) level in the anaerobic digester indicates the capability to counteract the acids concentration to maintain pH and to avoid the inhibition in the digestion process. TA should fall into the range of 1500 to 3000 mg CaCO_3_/L for optimal operation during AD process [18]. TA concentrations of the SE and CPTPS_1.5%_ treated CS were 1740 ± 20 and 1560 ± 10 and thus were found in the permissible range. Nitrogen is an essential nutrient for anaerobic bacterial culture for digestion process, and tolerable concentration of total ammonia nitrogen (TAN) ranges from 55 ± 11 to 150 mg/L for optimal digestion [28]. Concentration more than this permissible limit causes inhibition/toxicity for AD system. TAN concentration of CPTPS_1.5%_ treated CS was 133 ± 6.0 mg/L and located in allowable range for AD. Biodegradability (*B*_*d*_) is ultimate indicator for stable and optimal AD. *B*_*d*_ of 1.5% KOH at 60°C, SE, CPTPS_0.5%_, and CPTPS_1.5%_ treated CS were 57.21%, 51.16%, 55.66%, and 68.90%, respectively. *B*_*d*_ of CPTPS_1.5%_ treated CS was significantly improved 88.46%, 20.51%, 34.67%, and 23.81%, respectively, compared with untreated, thermal 1.5% KOH-60°C treated, SE treated, and CPTPS_0.5%_ treated CS, while EMY and *B*_*d*_ of combined treated CS with KOH and SE were only 258.8 mL/g_vs_ and 62.5%, respectively, which indicated significant differences compared with CPTPS_1.5%_ [22]. Therefore, considering the concentration of TA, TAN, and biodegradability after AD, CPTPS_1.5%_ treated CS digestion process was stable and the performance of methane production was very well.

### 3.5. Kinetics Analysis of Methane Production

**Figure 3:**
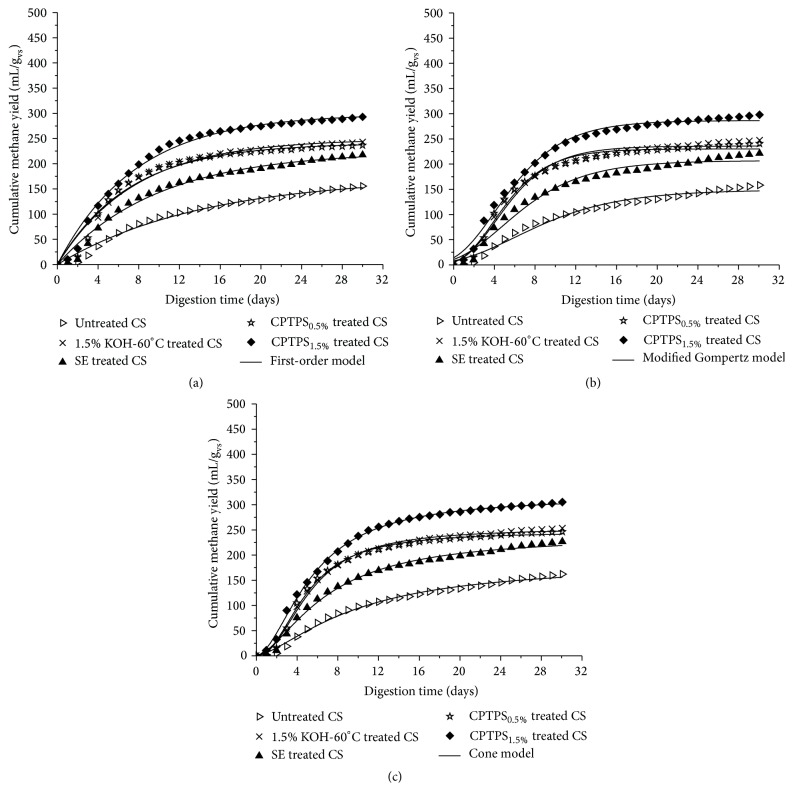
First-order, modified Gompertz, and Cone plots of cumulative methane yields of untreated, thermal KOH treated, SE treated, and CPTPS treated CS.

The simulation of cumulative biogas production of untreated, thermal KOH treated, SE treated, and CPTPS treated CS were analyzed by applying three kinetic models: first-order, modified Gompertz, and Cone models, and three plots are shown in Figure 3. *R* ^2^ values of first-order, modified Gompertz, and Cone models were ranged from 0.971 to 0.987, 0.977 to 0.991, and 0.993 to 0.998, respectively. *R* ^2^ of Cone model showed the best fit and well simulated the cumulative methane production of untreated, thermal KOH treated, SE treated, and CPTPS treated CS. These three models describe the different kinetics functions of AD process. So the following parameters of three kinetic models (*k*, *μ*_*m*_, *λ*, and *B*_0_) were estimated and presented in Table 3 for the kinetics of methane production.

According to prediction of first-order and Cone models, hydrolysis rate (*k*) value increased from untreated to pretreated CS; meanwhile, modified Gompertz model predicted that the lag phase time (*λ*) was dropped (0.45 ± 0.49 to 0.41 ± 0.23 day) from untreated to CPTPS_1.5%_ treated CS. Drop of *λ* implied a synergistic effect of CPTPS treatment on complex lignocellulosic structure deconstruction. Consequently, maximum methane production rate (*μ*_*m*_) value was increased from untreated to treated CS, and CPTPS_1.5%_ treated CS was looked high as 28.28 ± 1.24. Besides all these digestion parameters, methane production potential was estimated to be lower (281.6 ± 2.4 mL CH_4_/g_vs_) by modified Gompertz and slightly higher (303.5 ± 2.3 mL CH_4_/g_vs_) by Cone model of CPTPS_1.5%_ treated CS with respect to EMY value of 292.9 ± 3.8 mL CH_4_/g_vs_, while first-order model imitated 299.1 ± 4.1 mL CH_4_/g_vs_ and it was approximately near to EMY of CPTPS_1.5%_ treated CS. The results of kinetic parameters showed that CPTPS_1.5%_ pretreatment effectively deconstructed the complex nature of lignocellulosic structure and enhanced the methane yield as compared to other treatments. In spite of all these, EMY indicated that there was still a lot of space to do more research in future to improve the output yield of CS.

### 3.6. Changes of Physicochemical Structures

Physicochemical structural changes of untreated, KOH treated, SE treated, and CPTPS treated CS were examined by SEM, FTIR, and XRD.

#### 3.6.1. SEM Analysis

**Figure 4:**
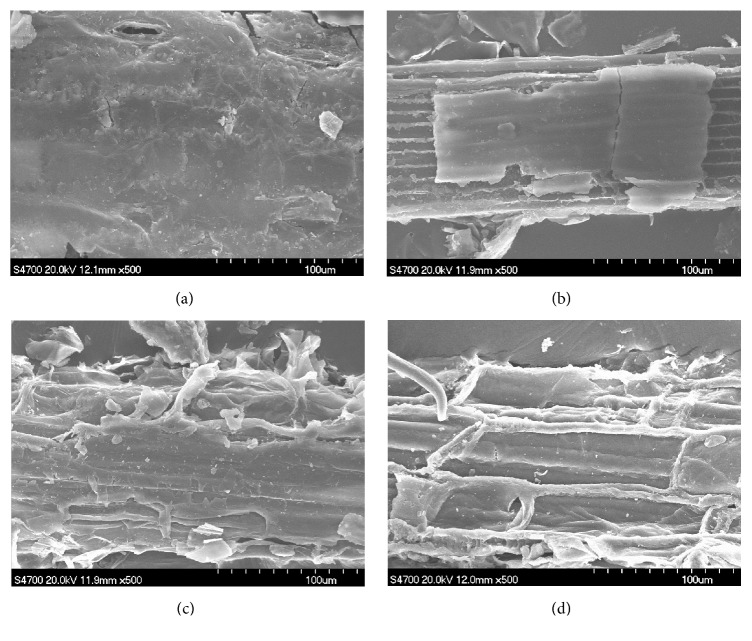
SEM images (500x) of untreated (a), SE treated (b), thermal KOH treated (c), and CPTPS_1.5%_ treated (d) CS.

The SEM images of untreated and treated CS are presented in Figure 4 for the surface morphological comparison. The morphology of untreated CS image “a” revealed more rigid and smooth surface than image “b” of SE treated CS, while image “b” was more dense and compact than image “c” of thermal KOH treated CS. On the other hand, image “d” of CPTPS_1.5%_ treated CS was extremely smashed, uneven, and ruptured as compared to images “a,” “b,” and “c.” It indicated that CPTPS treatment exposed and increased the surface area. Most of lignin and hemicellulose of CS were probably dissolved or broken down, and the cell wall structure was deconstructed.

#### 3.6.2. FTIR Analysis

**Figure 5:**
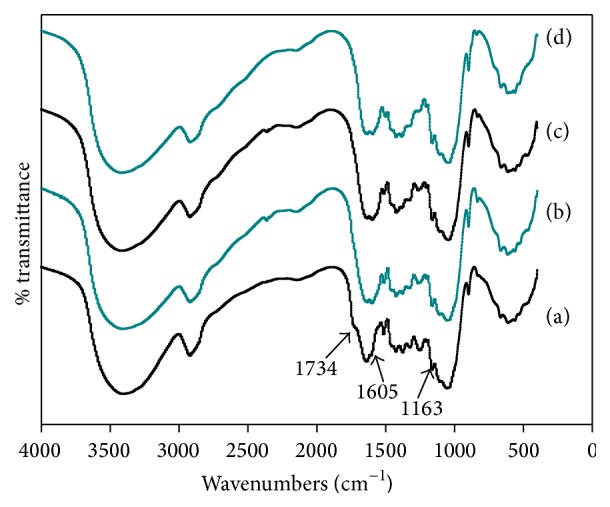
FTIR spectra of untreated (a), SE treated (b), thermal KOH treated (c), and CPTPS_1.5%_ treated (d) CS.

Changes in functional group structure of SE treated, thermal KOH treated, and CPTPS_1.5%_ treated CS with respect to untreated CS were examined by FTIR spectroscopy and the spectra are presented in Figure 5. All the samples of CS showed the similar trend, while the following bands showed some changes: 1734 cm^−1^, 1605 cm^−1^, and 1163 cm^−1^. The wavenumber around 1734 cm^−1^ band stands for carbonyl of hemicellulose, and reduction from untreated CS to CPTPS_1.5%_ treated CS was observed; in other words, copretreatment of thermal KOH and SE caused breakdown of ester bond in hemicelluloses and lignin. Beside this, the peak at 1605 cm^−1^ of thermal KOH-60°C treated CS was weaker than SE treated CS. It indicated more degradation of lignin in thermal KOH treatment than SE due to deconstruction of aromatic rings. Furthermore, peak at 1163 cm^−1^ increased in CPTPS_1.5%_ treated CS, compared to untreated, SE treated, and thermal KOH treated CS, because of changes in cellulose from crystalline to amorphous, lignin degradation or removal, and hemicellulose destruction. These indicators proved that CPTPS treatment was more effective to deconstruct the lignocellulosic structure for improving the anaerobic digestibility of CS.

#### 3.6.3. XRD Analysis

**Figure 6:**
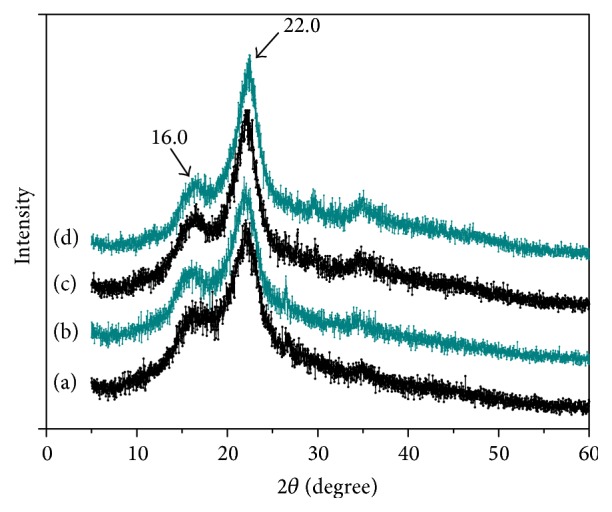
XRD patterns of untreated (a), SE treated (b), thermal KOH treated (c), and CPTPS_1.5%_ treated (d) CS.

XRD was applied to analyze the cellulose crystalline structures of untreated, thermal KOH treated, SE treated, and CPTPS_1.5%_ treated CS, and the spectra are presented in Figure 6. Similar pattern in XRD was observed, while changes appeared in 2*θ* of 22° and 18° due to crystalline variation. The collective effect of thermal KOH and SE is probably swelling the cellulose during the pretreatment process of CS, while cellulose crystal lattice may not change. The calculated CrI values of untreated, SE treated, thermal KOH treated, and CPTPS_1.5%_ treated CS were 48.14%, 57.49%, 58.07%, and 65.52%, respectively. Maximum CrI value was appeared in CPTPS_1.5%_ treated CS, compared to SE and thermal KOH treatments. It implied that CPTPS had synergistic effect to remove noncrystalline hemicellulose and lignin as compared to SE and thermal KOH. Therefore, CPTPS achieved the higher CrI values and improved the biodegradability of CS.

## 4. Conclusion

Three different types of pretreatment (KOH, SE, and CPTPS) were employed to CS for evaluation of their impacts on deconstruction of lignocellulosic structure, to improve the digestibility and enhance the methane yield. CPTPS_1.5%_ effectively altered the recalcitant nature and complex structure of lignocellulosic CS and increased the cumulative methane yield (292.9 mL/g_vs_) of CS by 20.50%, 34.66%, and 88.46%, compared with thermal KOH-60°C treated, SE treated, and untreated CS. CPTPS might be a potential pretreatment method to destroy the lignocellulosic structure and to improve the digestibility of CS for future AD industry.

## Figures and Tables

**Table 1:** Characteristics of CS and inoculum.

Parameter	CS	Inoculum
TS (%)^a^	93.99	7.25
VS (%)^a^	90.02	3.52
FS (%)^a^	3.98	3.73
VS/TS (%)	95.77	48.58
pH	ND	7.95
C (%)^b^	43.57	31.33
H (%)^b^	5.79	4.23
O (%)^b^	44.77	ND
N (%)^b^	1.16	2.85
C/N (%)	37.56	10.99
Cellulose (%)^b^	44.32	ND
Hemicellulose (%)^b^	33.54	ND
Lignin (%)^b^	8.28	ND

ND: not determined; CS: corn stover.

^a^Weight of sample; ^b^TS of sample.

**Table 2:** The concentration of TA, TAN, and biodegradability after AD of untreated, thermal KOH treated, SE treated, CPTPS_0.5%_ treated, and CPTPS_1.5%_ treated CS.

Samples	TA (mg CaCO_3_/L)	TAN (mg/L)	*B*_*d*_ (%)
Untreated CS	1085 ± 55	181.50 ± 8.50	36.57
1.5% KOH-60°C treated CS	1400 ± 20	173.50 ± 1.50	57.21
SE treated CS	1740 ± 20	195.00 ± 11.00	51.16
CPTPS_0.5%_treated CS	1420 ± 60	192.50 ± 5.50	55.66
CPTPS_1.5%_treated CS	1560 ± 10	133.00 ± 6.00	68.90

**Table 3:** Parameters of three models from AD of CS after different pretreatments.

Samples	First-order model			Modified Gompertz model				Cone model				EMY
	*B*_0_	*k*	*R* ^2^	*B*_0_	*µ*_*m*_	*λ*	*R* ^2^	*B*_0_	*n*	*k*	*R* ^2^	(mL/g_vs_)
Untreated CS	173.7 ± 5.6	0.070 ± 0.005	0.985	146.3 ± 3.1	9.62 ± 0.64	0.45 ± 0.49	0.977	176.7 ± 7.0	0.103 ± 0.007	1.51 ± 0.099	0.996	155.5
1.5% KOH-60°C treated CS	249.0 ± 5.2	0.132 ± 0.009	0.983	231.8 ± 2.3	27.58 ± 1.58	1.06 ± 0.25	0.987	243.0 ± 2.2	0.190 ± 0.004	2.17 ± 0.088	0.993	217.5
SE treated CS	224.5 ± 4.8	0.101 ± 0.006	0.983	203.4 ± 3.2	17.10 ± 1.16	0.40 ± 0.41	0.977	228.8 ± 5.2	0.146 ± 0.006	1.64 ± 0.093	0.996	243.2
CPTPS_0.5%_treated CS	240.9 ± 4.6	0.142 ± 0.009	0.971	226.2 ± 2.1	28.35 ± 1.64	0.98 ± 0.24	0.987	236.4 ± 2.0	0.202 ± 0.004	2.17 ± 0.087	0.998	236.6
CPTPS_1.5%_treated CS	299.1 ± 4.1	0.131 ± 0.006	0.987	281.6 ± 2.4	28.28 ± 1.24	0.41 ± 0.23	0.991	303.5 ± 2.3	0.183 ± 0.003	1.79 ± 0.047	0.998	292.9
